# Are dilution, slow injection and care bolus technique the causal solution to mitigating arterial-phase artifacts on gadoxetic acid–enhanced MRI? A large-cohort study

**DOI:** 10.1007/s00330-024-10590-1

**Published:** 2024-01-20

**Authors:** Sarah Poetter-Lang, Raphael Ambros, Alina Messner, Antonia Kristic, Jacqueline C. Hodge, Nina Bastati, Wolfgang Schima, Victoria Chernyak, Mustafa R. Bashir, Ahmed Ba-Ssalamah

**Affiliations:** 1 Department of Biomedical Imaging and Image-Guided Therapy, Medical University, General Hospital of Vienna (AKH), Waehringer Guertel 18-20, 1090 Vienna, Austria; 2 Department of Diagnostic and Interventional Radiology, Clinic Donaustadt, Vienna Healthcare Group, Vienna, Austria; 3 Department of Diagnostic and Interventional Radiology, Goettlicher Heiland Krankenhaus, Barmherzige Schwestern Krankenhaus, and Sankt Josef Krankenhaus, Vienna, Austria; 4 Department of Radiology, Memorial Sloan Kettering Cancer Center, New York City, NY USA; 5 Department of Radiology, Duke University Medical Center, Durham, NC USA

**Keywords:** Liver, Magnetic resonance imaging, Contrast media, Artifacts

## Abstract

**Objective:**

Arterial-phase artifacts are gadoxetic acid (GA)–enhanced MRI’s major drawback, ranging from 5 to 39%. We evaluate the effect of dilution and slow injection of GA using automated fluoroscopic triggering on liver MRI arterial-phase (AP) acquisition timing, artifact frequency, and lesion visibility.

**Methods and materials:**

Saline-diluted 1:1 GA was injected at 1 ml/s into 1413 patients for 3 T liver MRI. Initially, one senior abdominal radiologist, i.e., principal investigator (PI), assessed all MR exams and compared them to previous and follow-up images, as well as the radiology report on record, determining the standard of reference for lesion detection and characterization. Then, three other readers independently evaluated the AP images for artifact type (truncation (TA), transient severe motion (TSM) or mixed), artifact severity (on a 5-point scale), acquisition timing (on a 4-point scale) and visibility (on a 5-point scale) of hypervascular lesions ≥ 5 mm, selected by the PI. Artifact score ≥ 4 and artifact score ≤ 3 were considered significant and non-significant artifacts, respectively.

**Results:**

Of the 1413 exams, diagnostic-quality arterial-phase images included 1100 (77.8%) without artifacts, 220 (15.6%) with minimal, and 77 (5.4%) with moderate artifacts. Only 16 exams (1.1%) had significant artifacts, 13 (0.9%) with severe artifacts (score 4), and three (0.2%) non-diagnostic artifacts (score 5). AP acquisition timing was optimal in 1369 (96.8%) exams. Of the 449 AP hypervascular lesions, 432 (96.2%) were detected.

**Conclusion:**

Combined dilution and slow injection of GA with MR results in well-timed arterial-phase images in 96.8% and a reduction of exams with significant artifacts to 1.1%.

**Clinical relevance statement:**

Hypervascular lesions, in particular HCC detection, hinge on arterial-phase hyperenhancement, making well-timed, artifact-free arterial-phase images a prerequisite for accurate diagnosis. Saline dilution 1:1, slow injection (1 ml/s), and automated bolus triggering reduce artifacts and optimize acquisition timing.

**Key Points:**

*• There was substantial agreement among the three readers regarding the presence and type of arterial-phase (AP) artifacts, acquisition timing, and lesion visibility.*

*• Impaired AP hypervascular lesion visibility occurred in 17 (3.8%) cases; in eight lesions due to mistiming and in nine lesions due to significant artifacts.*

*• When AP timing was suboptimal, it was too late in 40 exams (3%) and too early in 4 exams (0.2%) of exams.*

**Supplementary Information:**

The online version contains supplementary material available at 10.1007/s00330-024-10590-1.

## Introduction

Gadoxetic acid–enhanced magnetic resonance imaging (GA-MRI) is increasingly used for the detection and characterization of focal liver lesions, as well as the assessment of liver function and predicting liver-related events [1, 2]. Optimal arterial-phase imaging is important for accurate lesion diagnosis. However, 5–39% of GA-MR images have arterial-phase (AP) artifacts that degrade image quality [3, 4]. Furthermore, suboptimal arterial-phase timing may preclude the detection of arterial phase hyperenhancement (APHE), which is required for non-invasive diagnosis of hypervascular lesions, in particular, hepatocellular carcinoma (HCC) [5]. Therefore, eliminating AP artifacts and acquiring appropriately timed AP images, e.g., using a fluoroscopic triggering technique, are paramount [5, 6]. Additionally, injecting a fixed 10 ml dose of GA (off-label use) may partially compensate for the relatively small volume and low gadolinium concentration [7, 8].

It is postulated that label-recommended intravenous administration of GA at a rate of 2 ml/s causes two types of artifacts: a temporary increase in the artery’s signal amplitude in k-space during image acquisition, which results in truncation artifacts (TA) [9, 10], and simultaneously acute fleeting elevation of peak GA blood plasma concentration which may activate central chemoreceptors causing patients to transiently hyperventilate, i.e., transient severe motion (TSM) [11, 12].

Two techniques, both considered off-label use, have been recommended to causally reduce both TA and TSM: firstly, a slow injection rate, e.g., 1 ml/s [13], and secondly, doubling the volume of contrast agent through 1:1 saline dilution [11]. Each maneuver independently doubles the bolus length. Thus, together, they quadruple the bolus duration, lowering AP peak plasma GA concentration [10, 11; 13], which may then fall below the threshold that triggers central chemoreceptors. By preventing hyperventilation, TSM is minimized [11, 12, 14]. Furthermore, combined dilution and slow injection prolong the bolus duration, thus providing a more uniform GA concentration during arterial-phase image acquisition, with homogeneous filling of k-space, reducing TAs [10, 11].

Recently, our group compared slow injection (i.e., 1 ml/s) of GA with versus without 1:1 saline dilution in a cohort of 112 patients, finding that we reduced non-diagnostic AP artifacts from 16 to 1% [15]. More interestingly, despite dilution and slow injection, there was an increase rather than drop in signal intensity, i.e., signal to norm (S_Norm_) or contrast to norm (C_Norm_), where liver SI was normalized to erector spinae muscle SI rather than air, i.e., signal to noise ratio (SNR). We presumed this was due to the stretched AP bolus, which allowed more time for signal acquisition [15]. In this confirmatory current study, using a cohort of 1413 consecutive patients, we wanted to validate the effect of dilution and slow injection to reduce AP artifacts in a large cohort. Using fluoroscopic triggering rather than the test bolus technique, we injected 1:1 saline-diluted GA at 1 ml/s, evaluating AP images for artifacts, acquisition timing, and hypervascular lesion visibility. Furthermore, we also wished to see how well TA and TSM could be distinguished [3, 4, 16–18] since visually, TSM artifacts extend beyond the abdominal wall, typically occurring in the phase-encoding direction [19] and cause blurring at organ boundaries [3, 4, 16–18], while TA or Gibbs phenomenon appears as a ringing artifact confined to the abdomen [9, 19].

## Materials and methods

### Patients

All patients gave written, informed consent for MRI. Our institutional ethics review board approved the retrospective data collection and analysis and waived the requirement for additional informed consent.

**Fig. 1:**
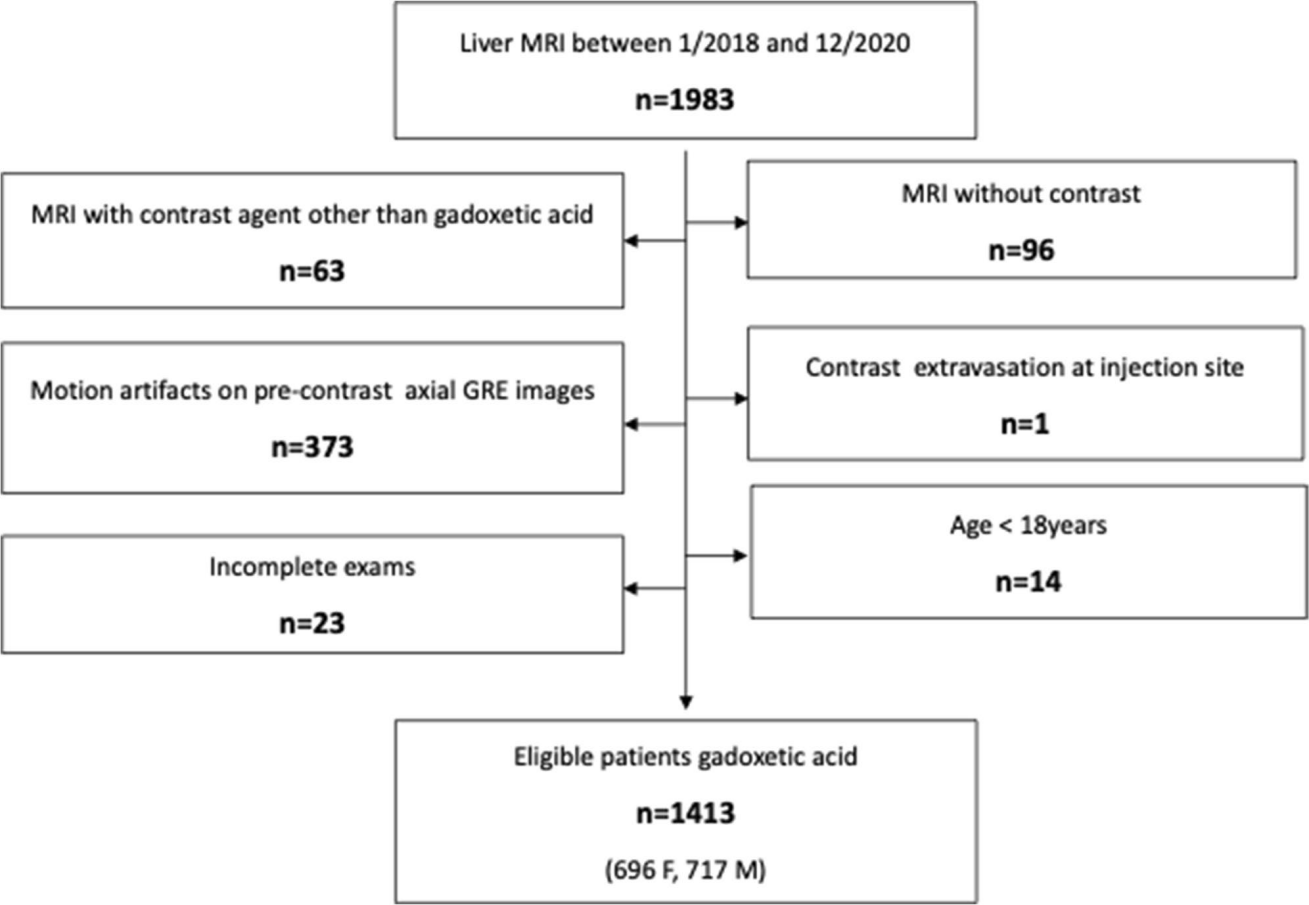
Flowchart showing the exclusion criteria

Between January 2018 and December 2020, we examined 1983 consecutive patients on a 3-T MRI system for known or suspected liver or pancreaticobiliary diseases. We excluded 570 patients for various reasons (see suppl), making the final study cohort 1413 patients (Fig. 1 flowchart). General patient and MRI-related characteristics, as well as factors possibly associated with AP artifacts, were recorded [4] [16, 20], including age, sex, weight, body mass index (BMI), history of lung or cardiac disease, any cancer, neuropsychiatric disorder, allergy to gadolinium-based contrast agents, MRI indication, presence and etiology of liver disease, Model For End-Stage Liver Disease (MELD) score and Child–Pugh class. To determine if a previous episode(s) of TSM predisposed to repeat TSM, the MR exams of the 461 patients who had undergone previous GA-enhanced liver MRI were reviewed for TSM presence and, if present, its severity.

### MR examination protocol

All patients underwent 3-T liver MR (Magnetom Prisma^Fit^, Siemens Healthineers, Erlangen, Germany) using a combined 60-element, phased-array abdominal coil, 30 channels anterior and 30 posterior. Unenhanced and contrast-enhanced dynamic imaging were performed using fat-suppressed T1-weighted three-dimensional gradient echo volumetric interpolated breath-hold examination (VIBE) Dixon sequences in the arterial, portal venous (70 s), transitional (300 s), and hepatobiliary (20 min) phases. The acquisition time per sequence ranged from 12 to 15 s (Table 1).

**Table 1:** MR protocol with exam parameters

Sequences	Slice orientation	Matrix	Voxel mm	FOV mm	SL mm	Phase Direction	TR ms	TE ms	FA Degree	Time s
T1 VIBE (FLASH 3D) in-phase	Axial	400 × 320	1.3 × 1.3 × 1.7	400	1.7–2	AP	4.4	2.66	20	15
T1 VIBE (FLASH 3D) opposed-phase	Axial	400 × 320	1.3 × 1.3 × 1.7	400	1.7–2	AP	4.4	1.33	20	15
T1 VIBE Dixon unenhanced	Axial	400 × 320	1.3 × 1.3 × 1.7	400	1.7–2	AP	4.4	1.33	20	15
T1 VIBE Dixon gadoxetic-enhanced (arterial and portal venous phases)	Axial	400 × 320	1.3 × 1.3 × 1.7		1.7–2	AP	4.4	1.33	20	15 × 3
T1 VIBE Dixon 5 min post contrast (transitional phase)	Axial	400 × 320	1.3 × 1.3 × 1.7		1.7–2	AP	4.4	1.33	20	15
DWI TSE-EP/ADC	Axial	400 × 134	1.5 × 1.5 × 5	400	5	AP	3400	38	90	314
T2 HASTE fatsat T2 HASTE	Axial Coronal	400 × 320 400 × 256	1.3 × 1.3 × 5 1.6 × 1.6 × 5	400 400	5 5	AP RL	1800 1800	154 154	150 143	88 94
T1 VIBE Dixon 20 min post contrast (HBP)	Axial	400 × 320	1.3 × 1.3 × 1.7	400	1.7–2	AP	4.4	1.33	20	15
T1 VIBE Dixon 20 min post contrast (HBP)	Coronal	243 × 320	1.4 × 1.4 × 1.5	450	1.5–2	RL	4.5	1.3	20	18

*HASTE* = half-Fourier acquisition single-shot turbo spin echo imaging; *DWI TSE-EP/ADC* = diffusion-weighted imaging turbo spin echo-echo-planar; *MRCP* = magnetic resonance cholangiopancreatography; *MIP* = maximum intensity projection; *GRE* = gradient echo, *VIBE* = volumetric interpolated breath-hold examination, *FOV* = field of view, *Voxel* = voxel size, *SL* = slice thickness, *TR* = repetition time, *TE* = echo time, *FA* = flip angle, time = acquisition time

### Contrast media injection techniques

Saline-diluted GA, 1:1 was administered as an intravenous bolus (a 10 ml fixed-dose in patients ≥ 50 kg or, if < 50 kg, at a dosage of 0.025 mmol/kg body weight (0.1 ml/ kg body weight)) through a 20- to 22-gauge antebrachial venous catheter. During the injection, continuous sagittal MR fluoroscopic images of the aorta were acquired using a rapid 2D gradient-echo technique. With a region of interest (ROI) placed over the aorta, either at the level of the celiac trunk or, if not seen, at the level of the diaphragm, the scanner identified the contrast bolus arrival. Once the specified signal threshold was exceeded, the machine automatically triggered the breath-hold command and started the acquisition within 6–8 s (see suppl).

### Qualitative image analysis

The standard of reference for focal liver lesion detection and characterization, if present, as well as lesion location, number, and size, was determined by the principal investigator (P.I., S.P.L.). Additionally, the PI recorded the presence of ascites (defined semi-quantitatively: none, mild, moderate, and severe [16]), cirrhosis (defined as present or absent), and/or pleural effusion (defined as present or absent) (see suppl).

Images were anonymized and then randomly analyzed visually on a dedicated picture archiving and communication system (PACS) workstation by three readers (A.K., R.A., and A.B. with 3, 5, and > 20 years of liver MRI experience, respectively). On AP images, each reader independently assigned ordinal numeric scores for artifact severity and acquisition timing. Before the study initiation, 20 cases not included in the study were used to train readers on timing, artifact type, and severity.

### Artifact severity

**Fig. 2:**
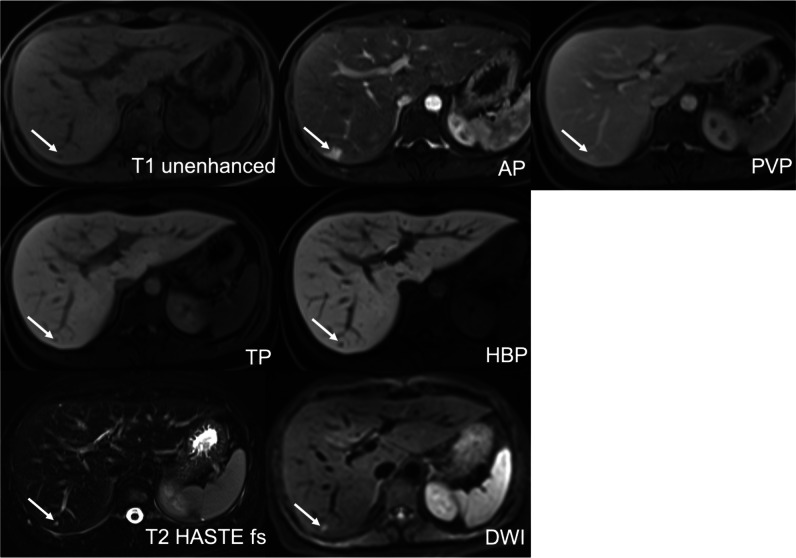
No AP artifacts, score 1, optimal timing, excellent lesion visibility, score 5: This 31-year-old woman was sent for MRI after an inconclusive ultrasound regarding a small focal liver lesion. A tiny T1-hypointense, markedly T2- and DWI-hyperintense lesion is seen in segment VI. On dynamic imaging, it shows strong corona enhancement in the AP, retained GA in the PVP, and pseudo-washout in the TP and HBP. There was no restriction on the ADC (not shown). The lesion is consistent with a flash-filling hemangioma

**Fig. 3:**
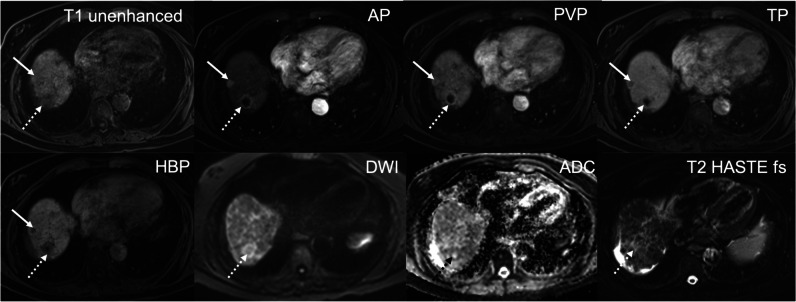
Minimal AP artifacts score 2, optimal timing and excellent lesion visibility, score 5: Minimal parallel bandlike AP artifacts confined to the abdomen consistent with TA: This 74-year-old man has a history of HCV cirrhosis. GA-enhanced MRI shows two types of hypervascular lesions in the arterial phase in liver segment VII/VIII, a tiny one with homogenous enhancement (non-rim APHE) and a larger lesion (dashed arrow) with rim-enhancement (rim APHE). The tiny lesion shows PVP washout and even more hypointensity in the TP and HBP. The larger lesion has a typical target appearance on both the HBP and DWI. The tiny lesion is an HCC (LR 5). The targetoid lesion (LR M) was histologically proven to be an atypical HCC (dashed arrow)

**Fig. 4:**
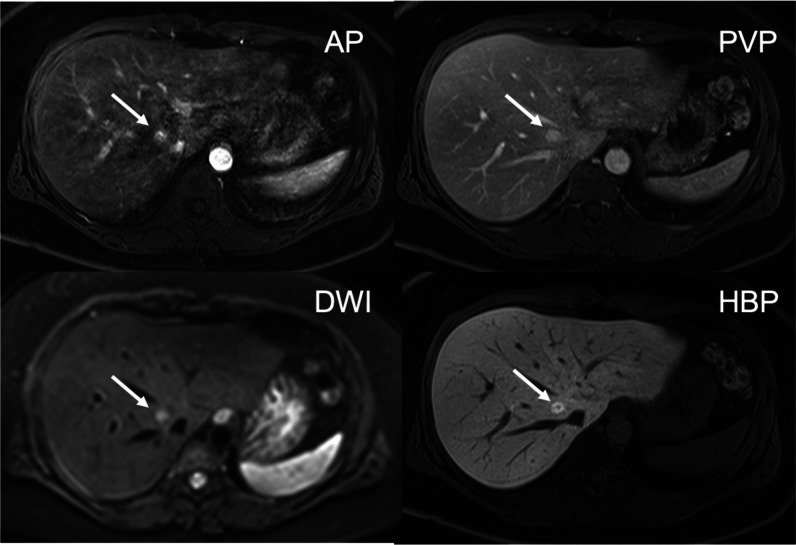
Moderate mixed AP artifacts, score 3, optimal timing, sufficient lesion visibility, score 3: Unsharp appearance of the structures and lesion margins and blurring at organ boundaries due to TSM (score 3) and multiple parallel stripes at high-contrast interfaces, especially in left liver lobe due to TA (score 2): This 33-year-old woman with NASH was referred for MRI because of questionable lesions on ultrasound. The AP shows a small lesion with hyperenhancement in liver segment VIII, despite AP artifacts. In the PVP, the lesion shows contrast retention, and on the 20-min HBP, it has more retention and a central scar. It is uniformly bright on DWI. The findings are consistent with a small FNH

**Fig. 5:**
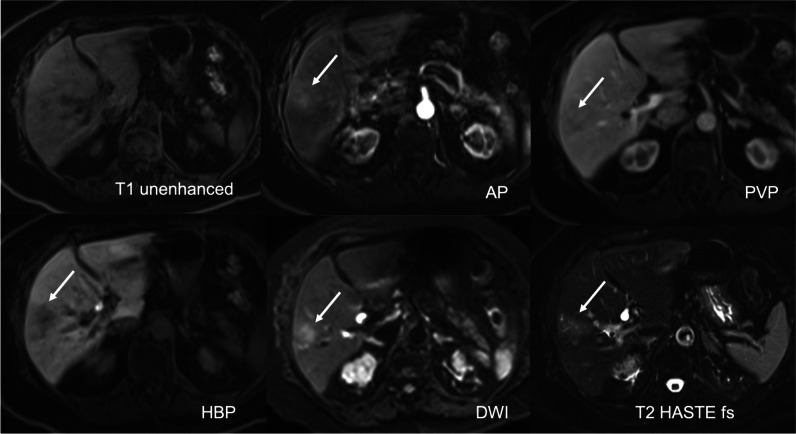
Severe mixed AP artifacts, score 4, optimal timing, uncertain lesion visibility, score 2: Multiple bright and dark lines parallel to the liver edge represent mild TA (score 2). Misregistration of the right kidneys and blurring at liver and kidney boundaries indicate severe TSM (score 4): This 94-year-old woman with Merkel cell carcinoma of the left cheek was sent for MRI to rule out liver metastases. A T2 mildly hyperintense lesion in liver segment V shows weak enhancement on the standard AP with washout in the hepatobiliary phase. DWI shows some restrictions centrally. A biopsy confirmed that this was a hypervascular metastasis. The diagnosis was uncertain due to the poor lesion visibility

**Fig. 6:**
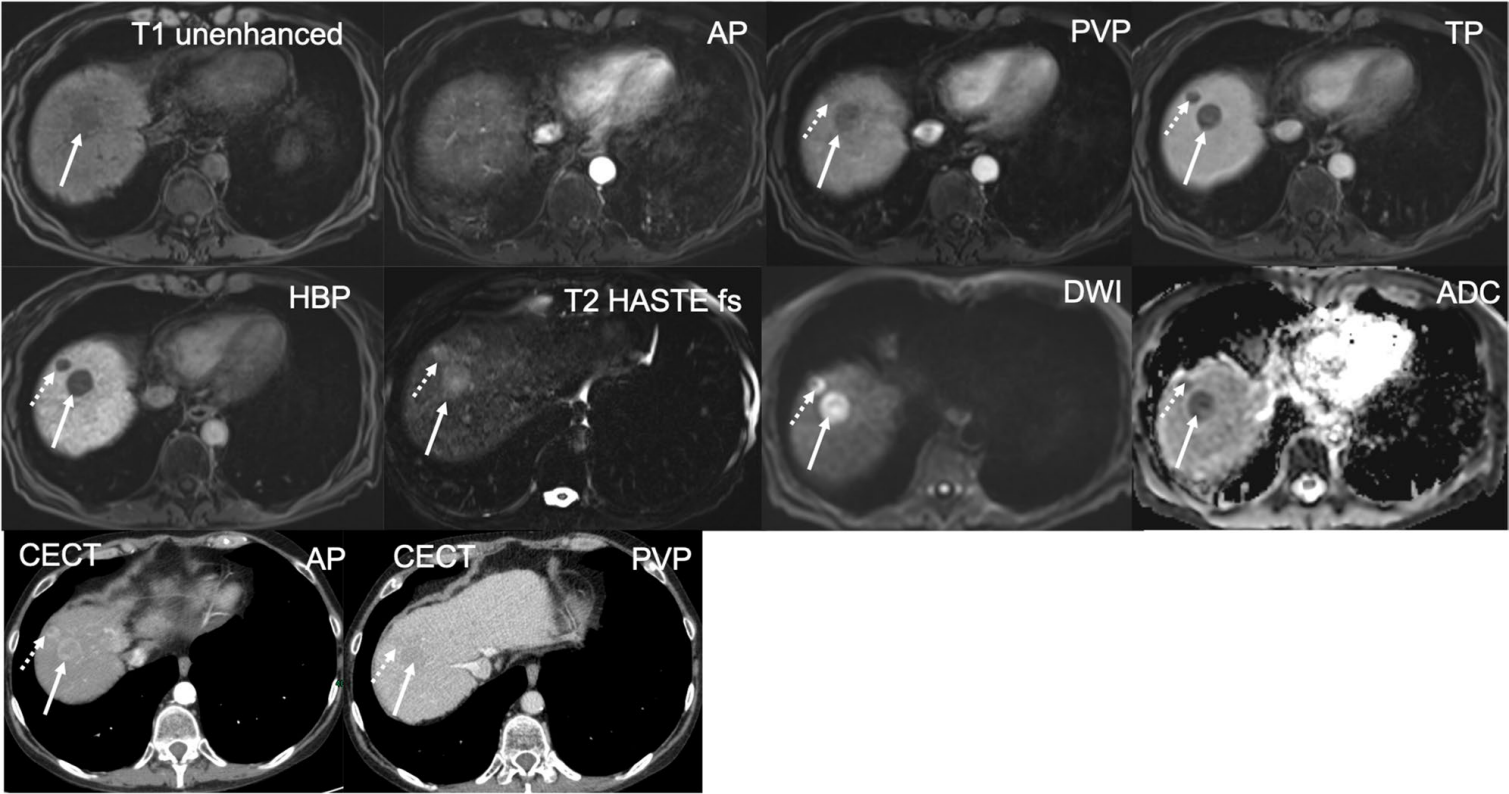
Severe TSM AP artifacts, score 4, suboptimal timing (too-early acquisition), poor lesion visibility, score 1: Blurring at boundaries between the liver and lung base indicating TSM. Neither lesion is assessable if compared with the HBP where they are now visible (score 1). This is a 79-year-old man with liver cirrhosis, Child–Pugh B and known HCC. On CECT, two HCC lesions with arterial phase hyperenhancement and PVP washout were seen in the liver dome. On MRI, the two HCC lesions are slightly T1 hypointense and T2 moderately hyperintense but barely seen in segment VIII of the liver on the AP due to too-early acquisition. In the PVP, both HCC lesions are hypointense and become better defined on the TP and HBP. Both lesions show diffusion restriction with high b values, b = 600

A priori*,* we set precise criteria for the three types of artifacts as follows:

Truncation artifacts (TAs) or ringing artifacts were defined as multiple bright or dark lines parallel to the edge of the interface or stripes at high-contrast interfaces that do not extend beyond the abdominal wall [17, 19]. TSM were considered any motion-related artifacts seen in the phase-encoding direction on AP images only. They cause structures to appear misaligned or blurred at organ boundaries, and they extend beyond the abdominal wall [3, 19]. Mixed artifacts meant that features of both TA and TSM were seen [19]. Then, the three readers independently assigned a score ranging from 1 to 5 [4, 20] as follows: (1) no artifacts; (2) minimal artifacts, without diagnostic impact; (3) moderate artifacts, with minimal diagnostic impact; (4) severe artifacts, but images still interpretable; and (5) non-diagnostic (Figs. 2, 3, 4, 5, and 6). Artifact severity scores for all three readers were then averaged and rounded to the nearest whole number, producing a mean AP artifact score. Significant artifacts were defined as ≥ 4, and non-significant artifacts as ≤ 3.

### Arterial-phase timing

Each reader independently evaluated the timing of AP image acquisition as follows [21]: 1 = too early (no contrast material in the hepatic artery); 2 = early arterial/arteriographic (contrast material in the hepatic artery but no portal vein or parenchymal enhancement); 3 = late hepatic arterial (strong enhancement of the hepatic artery, weaker enhancement of the portal vein than the hepatic artery, and no hepatic vein enhancement), 4 = too late (strong parenchymal enhancement or hepatic venous enhancement) [22]. The late hepatic arterial acquisition (#3) was considered optimal. All other acquisitions were considered suboptimal.

### Visibility of lesions with arterial phase hyperenhancement

Since our goal was to evaluate the diagnostic impact of AP image quality, the principal investigator (PI) selected only arterial phase hyperenhancement (APHE) lesions. If present, the PI chose the smallest and/or most difficult-to-see hypervascular lesion ≥ 0.5 cm in every MR exam. This was done to avoid mismatch. Among lesions with a clear diagnosis obtained from a review of the entire MR exam and previous and/or follow-up exams, the PI selected 449 hypervascular lesions. The PI judged a lesion hypervascular if clear arterial phase hyperenhancement (APHE) of the lesion was observed. Then the PI presented the single AP image that showed the lesion with APHE best to the three readers independently for scoring the confidence in lesion visibility on the following 5-point scale.

- 1 = APHE is not assessable.
- 2 = Presence of APHE is uncertain.
- 3 = Depiction of APHE is sufficient.
- 4 = Depiction of APHE is good.
- 5 = Depiction of APHE is excellent.

### Statistical analysis

Patient characteristics that are continuous variables (e.g., patient age, weight, and BMI) were presented as means ± standard deviations (SD) and ranges or median and quartiles as applicable, and those that are categorical variables (e.g., patient gender, presence of ascites, presence of pleural effusion, and presence of focal liver lesion) were presented as counts and percentages. The inter-rater variability was assessed using Fleiss’ kappa (κ) coefficient. The association of AP artifacts with ordinal features was assessed by Kendall’s rank correlation, and the association of AP artifacts with each binary feature was assessed by chi-square or Fisher’s exact test (depending on group size) after dividing AP artifacts into groups of “non-significant” (artifact grade 1 to 3) and “significant” (artifact grade 4 or 5). The association of AP artifact groups “non-significant” and “significant” with numeric features was assessed using Student’s t-test. For all statistical analyses, *p* < 0.05 was considered a statistically significant difference. All statistical analyses were performed using statistical software (R Studio, 1.4.1717, “Juliet Rose,” PBC) and IBM SPSS (version 26).

## Results

### Patient characteristics

Our cohort of 1413 patients included 717 (50.7%) males and 696 (49.3%) females. We have included only the initial study if patients had more than one MRI during the study period. The mean patient age was 55.8 ± 15.5 years (range: 18.0–90.0 years), mean BMI 25.0 ± 4.5 kg/m^2^ (range: 13.7–46.8 kg/m^2^). Thirty-one patients weighed < 50 kg, while 1382 patients weighed ≥ 50 kg. There were 1021 focal liver lesions in 1007 patients whose etiologies are listed in Table 2.

**Table 2:** Overview of patient characteristics

Patient characteristic	Number (%) or mean ± SD
Included patients	1413
Female patients	696 (49.3%)
Male patients	717 (50.7%)
Age (years)	55.8 ± 15.5
Height (m)	1.71 ± 0.94
Weight (kg)	73.2 ± 15.4
Body mass index (BMI, kg/m^2^)	25.0 ± 4.5
Liver cirrhosis	256 (18.1%)
Moderate or severe ascites	182 (12.9%)
Moderate or severe pleural effusion	121 (8.6%)
Patients with no focal liver lesion	406 (28.7%)
Patients with focal liver lesion	1007 (71.3%)
Total focal liver lesions	1021
Cyst	253 (24.8%)
Hemangioma (capillary and cavernous)	115 (11.3%)
FNH	89 (8.7%)
Adenoma	32 (3.1%)
Metastases (hypo-, and hypervascular)	197 (19.3%)
Cholangiocarcinoma CCA	16 (1.6%)
Miscellaneous	108 (10.5%)
HCC in non-cirrhotic liver	20 (2%)
Liver cirrhosis	
LR-2 observation	26 (2.6%)
LR-3 Observation	57 (5.6%)
LR-4 observation	19 (1.9%)
LR-5 observation	58 (5.6%)
Post-interventional lesion	31 (3.0%)

LI-RADS is used to describe lesions in patients at risk for HCC; all other lesion etiologies apply to non-cirrhotic patients

### Arterial-phase artifacts

AP artifacts occurred in 313 exams (22.2%): 36 (11.5%) TSM artifacts, 162 (51.8%) TAs, and 115 (36.7%) mixed artifacts. Only 16 (1.1%) exams had significant artifacts, i.e., score ≥ 4. Of these 13 were severe artifacts, and three were non-diagnostic artifacts (Table 3).

**Table 3:** Distribution of mean AP artifact scores and AP artifact types

Artifact Severity Score	Number (%) Exams	TSM	TA	Mixed
1 (None)	1100 (78.8%)	0	0	0
2 (Minimal)	220 (70.3%)	23 (10.5%)	155 (70.5%)	42 (19.1%)
3 (Moderate)	77 (24.6%)	8 (10.4%)	7 (9.1%)	62 (80.5%)
4 (Severe)	13 (4.1%)	3 (23.1%)	0 (0%)	10 (76.9%)
5 (Non-diagnostic)	3 (1.0%)	2 (66.7%)	0 (0%)	1 (33.3%)

A total of 313 of the 1413 exams (22.2%) had at least one type of artifact

Artifact score ≥ 4 was considered significant, artifact score ≤ 3 is considered non-significant

### Arterial-phase artifact scores in the context of AP artifact types

Of the 220 examinations with minimal artifacts, 155 (70.5%) were TAs, 23 (10.5%) were TSM, and 42 (19.1%) were mixed. Of the 77 examinations with moderate artifacts, seven (9.1%) were pure TAs, eight (10.4%) pure TSM, and 62 (80.5%) mixed. Of the 13 examinations with severe artifacts, none were pure TAs, three (23.1%) TSM, and 10 (76.9%) were mixed artifacts. Of the three exams made non-diagnostic by artifacts, two (66.7%) were TSM, and one (33.3%) a mixed artifact. A total of 36 TSM artifacts were observed: 23 (63.9%) minimal, eight (22.2%) moderate, three (8.3%) severe, and two (5.6%) non-diagnostic. TAs were primarily minimal 155 (95.7%), with just seven (4.3%) causing moderate artifacts and none being severe (Table 3).

### Inter-rater agreement for scores

For AP artifacts, there was substantial agreement among the three readers, with a Fleiss’ kappa of 0.725 (*p* < 0.001). Regarding the AP artifact type, the Fleiss’ kappa was also substantial 0.802 (*p* < 0.001). For both arterial-phase timing evaluation and lesion visibility, the agreement was substantial, with Fleiss’ kappas of 0.779 and 0.617, respectively (both *p* < 0.001).

### Factors affecting arterial-phase artifacts

When dichotomizing the exams into “significant” vs “non-significant” mean patient age was different between groups (64.6 ± 13.4 vs 55.7 ± 15.5 years, *p* = 0.022). However, there was no association with BMI (25.0 ± 4.6 vs 23.9 ± 2.7 kg/m^2^, *p* = 0.327) nor with 10 ml-fixed dose GA, if body weight ≥ 50 kg vs body weight-adjusted GA dose of 0.25 mmol/ml, if < 50 kg. There was also no association between artifact severity and gender (*p* = 06582), cirrhosis (*p* = 0.751), ascites (*p* = 0.241), or pleural effusion (*p* = 0.497).

We reviewed the exams of 461 (32.6%) cohorts who had a prior MRI. Of these, 445 (96.5%) had no significant artifacts on either prior or index exams. From the remaining 16 patients who had significant artifacts on the index exam, 13 (81.2%) of them had also had artifacts on the prior study (*p* < 0.001).

### Qualitative assessment of arterial-phase timing

Arterial-phase timing was optimal in 1369 exams (96.8%) and suboptimal in 44 (3.2%) (too early in four exams (0.2%) and too late in 40 exams (3%)). Comparing exams with “optimal” vs “suboptimal” phase-timing, there was no significant difference in mean BMI between the two groups (suboptimal: 25.5 ± 5.4 kg/m^2^ vs optimal: 24.9 ± 4.5 kg/m^2^, *p* = 0.100). Cirrhosis (14.8% vs 10.3%, *p* = 0.038) and ascites (20.5% vs 10.5%, *p* = 0.005) occurred significantly more frequently on “suboptimal” vs “optimal” exams.

### Qualitative evaluation of arterial-phase hypervascular lesion visibility on MRI

From 1007 patients with a total of 1021 lesions, only 449 hypervascular (APHE) lesion (size range 5–156 mm, mean ± SD, 17.2 mm ± 17.2 mm) in 449 (44.6%) patients were selected by the principal investigator for confidence-rating. Inter-reader agreement was substantial, with a kappa of 0.609 (*p* = 0.001). Four hundred thirty-two (96%) lesions with arterial phase hyperenhancement were at least sufficiently seen (score 3 (*n* = 12, 3%), score 4 (*n* = 129, 30%), or score 5 (*n* = 291, 67%)). Of the 17 lesions that were not assessable (*n* = 4, score 1) or uncertain (*n* = 13, score 2), eight lesions could not be seen due to suboptimal arterial phase and nine lesions due to significant artifacts (Figs. 2, 3, 4, 5, and 6).

All of the 17 (3.8%) hypervascular lesions that were not clearly seen during the AP were known as lesions with APHE because they had been seen on other sequences and/or previous exams.

Of the 449 lesions, 433 (96.4%) were in exams with non-significant (artifact score ≤ 3) and 16 (3.6%) in exams with significant (artifact score ≥ 4) AP artifacts. In exams with non-significant artifacts, lesion visibility was good or excellent in 406 (93.8%), sufficient in 19 (4.4%), and not assessable or uncertain in eight (1.8%). In MRIs with significant artifacts, lesion conspicuity was good or excellent in only seven (43.8%) and not assessable or uncertain in nine (56.2%). This difference was statistically significant (*p* < 0.001). Further, lesion visibility scores and AP artifact scores were correlated with better lesion visibility when artifact scores were lower (*p* < 0.001). There was a statistically significant difference between phase-timing evaluation and lesion visibility (*p* < 0.001). No significant correlation was found between lesion visibility and BMI (*p* = 0.085). No significant correlation was found between visibility and size (*p* = 0.690) or age (*p* = 0.727). Factors affecting lesion visibility are summarized in Table 4 and supplementary.

**Table 4:** Factors affecting a total of 449 APHE lesion visibility

		Excellent/good/ sufficient	Uncertain/ not assessable	*p* value
*Significant Artifacts*	**Yes**	7 (1.6%)	9 (52.9%)	** < 0.001**
	**No**	425 (98.4%)	8 (47.1%)	
*Optimal late arterial phase*	**Yes**	392 (90.7%)	8 (47.1%)	** < 0.001**
	**No**	40 (9.3%)	9 (52.9%)	
*Gender*	**Male**	207 (47.9%)	9 (52.9%)	0.507
	**Female**	225 (52.1%)	8 (47.1%)	
*Age*	**(years)**	56.6 ± 14.7	58.1 ± 19.0	0.690
*BMI*	**(kg/m**^**2**^**)**	25.7 ± 4.6	28.1 ± 5.7	0.085
*Cirrhosis*	**Yes**	133 (30.8%)	6 (35.3%)	0.693
	**No**	299 (69.2%)	11 (64.7%)	
*Ascites*	**Yes**	92 (21.3%)	7 (41.2%)	0.071
	**No**	340 (78.7%)	10 (58.8%)	
*Pleural effusion*	**Yes**	66 (15.3%)	3 (17.6%)	0.734
	**No**	366 (84.7%)	14 (82.4%)	
*Lesion size*	**5–12 mm**	220 (50.9%)	8 (47.1%)	0.727
	** > 12 mm**	212 (49.1%)	9 (52.9%)	

Bolded *p* values represent statistically significant results

## Discussion

In this large cohort single-center study, we could reduce severe or non-diagnostic AP artifacts to only 1.1% by combining slow injection (1 ml/s) and 1:1 saline dilution of GA. This technique largely eliminates GA’s major drawback, i.e., arterial-phase artifacts, which range from 5 to 39% [23–25], impacting the diagnosis of lesions with arterial phase hyperenhancement, in particular HCC.

Besides artifacts, improper AP timing contributes to poor detection of hypervascular lesions. Therefore, we used automatic fluoroscopic triggering to optimize scan acquisition timing. In agreement with our findings, by injecting GA at 1 ml/s, Goshima et al found that the optimal scan delay for imaging AP hypervascular (APHE) HCCs was 7–12 s after peak aortic enhancement [26]. Furthermore, by administration of fixed-dose (10 ml) GA in patients with ≥ 50 kg body weight, we may partially have compensated for its lower gadolinium concentration [27, 28]. Additionally, the major advantage of 3 T versus 1.5 T is a nearly 1.5-fold gain in signal-to-noise ratio, which can support higher spatial resolution [28, 29]. Finally, like Poetter-Lang et al, we may have improved hypervascular (APHE) lesion visibility by increasing signal intensity and contrast ratio through dilution and slow injection [15]. These results are considered to be confirmatory to the above-mentioned controlled study, i.e., diluted (D) vs non-diluted (ND) exams and using quantitative criteria, including SI measurements [15]. Furthermore, our qualitative assessment criteria for grading artifacts were previously validated [4, 16, 20, 30].

Contrary to strategies to bypass AP artifacts, e.g., performing multiple rapid arterial phases and using modern techniques with free breathing [10, 13, 16, 22, 30], we chose combined dilution and slow injection of GA as a causal solution to reduce both TA and TSM. Slow injection doubled the bolus transit time of GA [6]. Saline dilution 1:1, by doubling the bolus volume, again doubled bolus transit time [10]. Therefore, the cumulative effect was quadrupled bolus transit time of GA [28], which reduced the mismatch between a short transit time and a relatively long image acquisition time. This reduced TAs [31] by making a more uniformly shaped bolus during image acquisition, resulting in a more homogenous k-space. Moreover, just as McQueen et al concluded that transient severe hyperventilation was due to immediate activation of peripheral chemoreceptors [32], we hypothesized that by combining dilution and slow injection, we would minimize TSM frequency through lowered peak plasma GA concentration below the threshold that triggers central chemoreceptors [11, 15]. The absence of TSM in children and those under sedation, perhaps due to incomplete development and suppression of chemoreceptors, respectively, may also support this theory [12]. Once chemoreceptors have matured, it may no longer be possible to willingly mitigate TSM through education and training, as has been observed empirically [23].

Although artifacts were still observed on 22.2% of exams, well within the reported range of 5–39% [23–25], most were judged to have no significant diagnostic impact. Furthermore, this figure included TAs (11.1%), which tend to be trivial and therefore underreported in the literature [6, 10, 19, 28]. Our high number of non-significant artifacts, despite dilution and slow injection, may be attributed to relatively long AP acquisition times, ranging between 12 and 15 s to maximize spatial resolution [9]. However, the influence of shorter or longer scanning times on TSM remains controversial [24, 25].

We chose dilution and slow injection rather than acquiring multiple arterial-phase images because the higher temporal resolution needed for rapid acquisition reduces spatial resolution, which may adversely impact liver lesion conspicuity [28]. Similar arguments apply to free-breathing techniques. Recent rapid-acquisition sequences, e.g., GRASP-VIBE, may generate artifacts that interfere with lesion visibility and generate hundreds of redundant images that are not used diagnostically [28]. Moreover, these techniques are not widely available, particularly on older MR systems [29].

The GA dose is controversial. TSM has been reported to occur 1.5 times more often (i.e., 15%) in patients who received a 20-ml rather than 10-ml fixed dose of GA [24]. However, in several studies extrapolating data from various sites, no dose-dependent relationship between GA dose and TSM frequency could be confirmed [3, 16, 20]. We found no correlation between body weight and TSM frequency, comparing our patients who received fixed-dose versus weight-based 0.025 ml/l dose of GA.

As in other studies, we found that prior episodes of TSM were significantly associated with the occurrence of TSM [3, 20, 23]. We also observed that older age was associated with more frequent AP artifact occurrence, similar to Shah [33]. However, no other publication confirmed this association [4, 25]. We found no correlation between chronic liver disease or cirrhosis and AP artifact occurrence, in line with previous studies [11, 13, 34]. Importantly, our results are in line with three retrospective analyses of conditions known to affect breath-hold ability, i.e., pleural effusions, ascites, and cardiac disorders, which also found no correlation with TSM [3, 20, 24].

Despite automatic fluoroscopic triggering, acquisition timing was suboptimal in 3.2% of cases, either too late or too early. This affected lesion visibility as we missed eight of 449 AP hypervascular lesions (1.8%). We found that risk factors for suboptimal arterial-phase timing included cirrhosis and ascites. We attribute this to hemodynamic changes related to liver cirrhosis [35]. No relationship was found between AP delay and AP artifacts.

To our knowledge, ours is the first study to evaluate the role of AP timing, in addition to artifacts, in hypervascular lesion visibility.

Our study had several limitations. Firstly, it was a single-center study with inherent potential bias, though we believe our results are valid because of the large, consecutively enrolled cohort. Secondly, we have no control or comparison group, i.e., diluted vs non-diluted group. This was done in the previous Poetter-Lang et al study [15], which we have now validated in a large cohort study. Thirdly, although there are minimal differences between the initial study by Poetter-Lang et al and the present study, including the injected dose (either fixed-dose or per-kilogram dose based on body weight), the recent study can be considered to validate our previous findings since we found that the artifact frequency was independent of the injected dose. Fourthly, the strict separation of TSM and TA is unrealistic when they occur simultaneously. Indeed, contamination by mixed artifacts could have led to errors in estimating TAs; however, we believe this risk was minimized by standardizing our image analysis criteria a priori*.* Fifthly, the risk of potential contamination should not deter radiologists from using dilution. There have been no reports of such in the literature [11, 15]. Lastly, the additional time needed to dilute GA is now a moot point as a new power injector, which simultaneously injects contrast media and saline, is now available. Thus, minimizing AP artifacts can be achieved automatically and without any risk of contamination or delay.

In conclusion, combined dilution and slow injection of gadoxetic acid with fluoroscopic triggering is a potential solution to optimize the timing of AP acquisition and to reduce diagnostically significant AP artifacts. Improved k-space homogeneity reduces TAs and lowering peak plasma GA concentration obviously prevents triggering central chemoreceptors to induce TSM artifacts.

### Supplementary Information

Below is the link to the electronic supplementary material.

Supplementary file1 (PDF 77 KB)

## Abbreviations

AP: Arterial phase
APHE: Arterial-phase hyperenhancement
BMI: Body mass index
CNR: Contrast to noise ratio
GA: Gadoxetic acid
GA-MRI: Gadoxetic acid–enhanced magnetic resonance imaging
HASTE: Half-Fourier single-shot turbo spin-echo
HBP: Hepatobiliary phase
ICC: Intraclass correlation coefficient
IV: Intravenous
MRI: Magnetic resonance imaging
PI: Principal investigator
ROI: Region of interest
SD: Standard deviation
SI: Signal intensity
SNR: Signal-to-noise ratio
TA: Truncation artifact
TSM: Transient severe motion artifact
VIBE: Volumetric interpolated breath-hold examination

## Declarations

### Guarantor

The scientific guarantor of this publication is Dr. Ahmed Ba-Ssalamah.

### Statistics and biometry

Dr. Michael Weber kindly provided statistical advice for this manuscript.

Alina Messner, one of the authors, has significant statistical expertise.

### Cohort overlap

None of the study subjects or cohorts have been previously reported.

### Methodology

• retrospective

• observational

• performed at one institution
