# Supplementary material for: Are dilution, slow injection and care bolus technique the causal solution to mitigating arterial-phase artifacts on gadoxetic acid–enhanced MRI? A large-cohort study
Source: Eur Radiol. 2024 Jan 20;34(8):5215–27. doi: 10.1007/s00330-024-10590-1 (PMC11254987; doi:10.1007/s00330-024-10590-1)
Supplement: Supplementary file 1 — Supplementary file1 (PDF 77 KB) [file 330_2024_10590_MOESM1_ESM.pdf]

Are dilution, slow injection and care bolus technique the causal solution to mitigating arterial-phase artifacts on gadoxetic acid-enhanced MRI? A large-cohort study

Electronic Supplementary Material (ESM)

Eur Radiol (2024) Poetter-Lang S, Ambros R, Messner A et al.

MATERIALS AND METHODS

Patients Excluded

Patients unable to give consent or who had MRI contraindications (e.g., cardiac pacemakers, known adverse events related to gadolinium chelates, and/or terminal renal failure as indicated by a glomerular filtration rate < 30 mL/min) were not included. Furthermore, 96 and 63 patients were excluded because they had non-contrast MR exams or received a contrast agent other than gadoxetic acid, respectively. One patient was excluded because of contrast extravasation into local tissues. We also excluded patients with a) motion artifacts on precontrast axial 3D T1-weighted spoiled GRE images, on the assumption that the artifacts seen on post-contrast images were not solely gadoxetic acid injection-related artifacts (n=373), b) incomplete exam (n=23), and/or c) age <18 years (n=14).

MR Examination Protocol

The pre-scan normalization filter provided by the vendor was activated in all sequences to homogenize the SI, except when performing multi-echo sequences (ME) for iron quantification. This sequence was acquired using the body coil integrated into the magnet.

VIBE Dixon sequence parameters were: TR 4.4 ms, TE 1.33 ms, phase direction AP, and flip angle 20°. Depending upon the patient size, slice thickness (1.7 to 2 mm), matrix size (400x320), FOV (400 mm) and number of slices (90 to 120) varied. Parallel imaging with an acceleration factor of two was used. Matrix and slice thickness given are for the reconstruction using zero interpolation. Axial T1-weighted VIBE images, covering the whole liver during end-expiratory breath hold, were obtained pre-contrast and during arterial, portal venous (70 s), transitional (300 s) and hepatobiliary (20 min) phases. In addition, coronal plane HBP images were obtained at 20 seconds, using Abdomen Dot Engine, where the sequence duration is adapted to the breath hold capability of the patient and patient size. Our k-space ordering was sequential.

Contrast Media Injection Techniques

Saline-diluted gadoxetic acid, 1:1 (i.e., total volume of 20 mL), was administered as

an intravenous bolus [a 10 ml fixed-dose in patients ≥ 50 kg or, if < 50 kg, at a dosage of 0.025 mmol/kg body weight (0.1 ml/ kg body weight)] through a 20- to 22-gauge antebrachial venous catheter, at an injection rate of 1ml/s followed by a 20-mL saline flush at the same rate. All injections were performed using a commercially available power injector.

During the injection a sagittal MR fluoroscopic-like image of the aorta was acquired using a rapid 2D gradient-echo technique. The technologists placed a region-of interest (ROI) over the aorta at the level of the celiac trunk, but when it was not clearly identified, they placed the ROI at the level of the diaphragm, the scanner identified the contrast bolus arrival. Once the specified signal threshold (e.g., 20% above baseline) was exceeded, the machine automatically triggered the breath hold command and started the acquisition. This automatic fluoroscopic bolus tracking and triggering software, offered by Siemens, is known as CARE Bolus. It allowed the timing of arterial-phase image acquisition to be tailored to each individual. Patient breathing instructions were given using an automated voice recording. The mean time to arterial-phase imaging was 30.3 s (range, 26–38 s). Given an approximate 6-8 seconds delay for providing breath hold instructions, the 3D GRE was commenced an average of 25.2 ± 6 4.1 seconds from the beginning of contrast administration.

Qualitative Image Analysis

An abdominal radiologist with >10 years of experience, the principal investigator, (PI), who did not participate in the analysis, assessed the whole, multi-sequence MRI for all study patients, comparing them to previous and follow-up images, including CTs, if available, as well as the radiology report on record. To avoid mismatch on images that had multiple lesions, the PI selected the images to be presented to each reader. Furthermore, the PI intentionally chose the most difficult-to-see AP lesions. When available, histologic diagnosis was recorded. Otherwise, radiologic diagnoses were based upon characteristic imaging features, underlying diseases, and interim change. All this data served as the standard of reference for lesion detection and characterization, allowing the PI to determine, if present, the location, number and size of focal liver lesions. This was particularly useful to confirm that a lesion was hypervascular or showing arterial phase hyperenhancement (APHE) when the AP images were nondiagnostic, as all these exams were correlated with previous

contrast enhanced CT or MRI other than the index exam to confirm these lesions had APHE.

While APHE is frequently used within the LI-RADS framework to characterize specific features of liver lesions, the term itself isn't exclusive to LI-RADS. Here, we use it in the broader sense, i.e., to describe the observation of hyperenhancement during the arterial phase of contrast imaging in either cirrhotic or non-cirrhotic patients. Therefore, we have applied the term to other lesions, e.g.., FNH, adenoma, hemangioma etc.

Results

Patient characteristics

Of our 1413 patients, 406 (28.7%) had no focal liver lesions and 1007 (71.3%) had focal liver lesions, of which 367 were malignant (35.9%) and 654 (64.1%) were benign, making a total of 1021 lesions (Table 2). There was histological proof in 98 (9.6%) hepatic lesions. The remaining 923 (90.4%) lesions were diagnosed based upon characteristic imaging findings and/or interim growth or stability on 1–3-year follow-up examinations.

There were 830 lesions in non-cirrhotic patients and 191 lesions in cirrhotic patients. In non-cirrhotic patients, there were 197 metastases (23.7%), 20 HCC (2.4%), 16 CCA (1.9%), 253 cysts (30.5 %), 115 hemangiomas (13.9%), 89 FNH (10.7%), 32 adenoma (3.9%) and 108 miscellaneous lesions (13.0 %), such as segmental cholangitis/abscess, THIDs, etc.

In the patients with liver cirrhosis, there were 191 lesions, including 26 LR-2 (13.6 %), 57 LR-3 (29.8%), 19 LR-4 (10.0%), and 58 LR-5 observations (30.0%), as well as 31 post-interventional lesions (16.2%).

Qualitative Measurement of Arterial-phase timing

When dichotomizing exam phase-timing evaluation into “optimal” and “suboptimal” (i.e., “too early” and “too late”) the mean patient weight was significantly lower in those with optimal versus suboptimal exams (72.8 ± 14.9 kg vs. 76.9 ± 18.4 kg,

p=0.007). There were significantly more males than females with “suboptimal” exams (13.3% vs 8.9%, p=0.009). No correlation was found between phase-timing evaluation and cirrhosis grade (p=0.326, BMI (p=0.254) or age (p=0.085).

Qualitative Evaluation of Hypervascular (APHE) Lesion Visibility on MR Images

From 1007 patients, and a total of 1021 lesions only 449 hypervascular lesions (size range 5-156 mm, mean± SD, 17.2 mm ±17.2 mm) in 449 (44.6%) were selected by the principal investigator for confidence-rating. Inter-reader agreement was substantial with a kappa of 0.609 (p=0.001). Of the 449 hypervascular lesions, 315 were in non-cirrhotic livers, including 32 adenomas (7.1%), 89 FNHs (19.8%), 115 hemangiomas (25.7%), 59 hypervascular metastases (13.1%), and 20 HCCs (4.5%). In the 134 cirrhotic livers, we found 57 LR- 3.
